# Omicron Variant of SARS-CoV-2: An Indian Perspective of Vaccination and Management

**DOI:** 10.3390/vaccines11010160

**Published:** 2023-01-11

**Authors:** Vivek P. Chavda, Pankti Balar, Dixa Vaghela, Hetvi K. Solanki, Akta Vaishnav, Vivek Hala, Lalitkumar Vora

**Affiliations:** 1 Department of Pharmaceutical Chemistry, L. M. College of Pharmacy, Ahmedabad 380009, Gujarat, India; 2 Pharmacy Section, L. M. College of Pharmacy, Ahmedabad 380009, Gujarat, India; 3 School of Pharmacy, Queen’s University Belfast, 97 Lisburn Road, Belfast BT9 7BL, UK

**Keywords:** omicron, vaccination, diagnosis, hesitancy, India, monkeypox, tomato flu

## Abstract

Omicron variants have highly influenced the entire globe. It has a high rate of transmissibility, which makes its management tedious. There are various subtypes of omicron, namely BA.1, BA.2, BA.3, BA.4, and BA.5. Currently, one omicron subvariant BF.7 is also immersed in some parts of India. Further studies are required for a better understanding of the new immersing SARS-CoV-2 subvariant of the omicron. They differ in the mutation of the spike proteins, which alters their attachment to the host receptor and hence modifies their virulence and adaptability. Delta variants have a great disastrous influence on the entire world, especially in India. While overcoming it, another mutant catches the pace. The Indian population is highly affected by omicron variants. It alters the entire management and diagnosis system against COVID-19. It demanded forcemeat in the health care system, both qualitatively and quantitively, to cope with the omicron wave. The alteration in spike protein, which is the major target of vaccines, leads to varied immunization against the subvariants. The efficacy of vaccines against the new variant was questioned. Every vaccine had a different shielding effect on the new variant. The hesitancy of vaccination was a prevalent factor in India that might have contributed to its outbreak. The prevalence of omicron, monkeypox, and tomato flu shared some similarities and distinct features when compared to their influence on the Indian population. This review emphasizes the changes omicron brings with it and how the Indian health care system outrage this dangerous variant.

## 1. Introduction

Coronavirus disease 2019 (COVID-19) has carried more complications for public health. Later, a new severe acute respiratory syndrome coronavirus 2 (SARS-CoV-2) variant with an amino acid mutation in the spike protein was reported to increase the severity of the disease [1,2]. The World Health Organization (WHO) divides variants into different categories, such as variants of concern (VOCs), variants of interest (VOIs), and variants under monitoring (VUMs) [3,4]. VOCs incorporate alpha, beta, gamma, and delta variants of SARS-CoV-2 [5]. In November 2021, a new variant named omicron was found by the WHO and is also included under the category of VOCs [6]. The delta (B.1.617.2) variant was first recognized in India as a VOC in October 2020. It is more contagious than previous alpha and beta previous variants [4,7]. Mutations in its receptor binding domain, namely, T478K and L452R, increase the transmission rate and infectivity rate of the delta variant [8]. Furthermore, the acquired K417 N mutation in the spike protein of delta leads to sublineages AY.1 and AY.2, which are called Delta Plus [9]. Data show that delta plus increased the infection rate by spreading 60% faster than the Delta variant because of its high affinity toward lung cells [10]. Delta cases decline in India due to the implementation of public health and weather conditions. Specifically, in India, the delta has a higher resistance to the drug used against COVID-19 and a potential reduction in the monoclonal antibody response [11]. Omicron was more severe, with a high number of mutations, almost 15 spike protein mutations, which immediately increased the risk of disease worldwide [12]. The prevalence of infection was 50% for beta, 80% for delta and 90% for omicron variant [13]. The original strain of Omicron was more transmissible than the delta and beta viruses and spread faster than other variants of SARS-CoV-2. It has revealed a high number of nonsynonymous mutations as well as spike mutations; overall, 60 substitutions and deletions in the S protein have been found in the omicron variant. Among five VOCs, the spike mutation found in omicron was 3–4 times higher [3,14,15]. Various organizational health consequences of omicron and other variants of SARS-CoV-2 have been observed. Balancing the right of nursing home residents to communication and their safety was very difficult. By protecting the right of elderly people to social contact, managing distanced interaction, and clinical risk management, the risk of infection is decreased [16,17]. Infect, veterinary vaccines are also approved and many under clinical develop ments [18,19].

The mutation increases the binding of the spike protein with angiotensin-converting enzyme 2 (ACE2), which leads to higher transmission and pathogenicity and reduces the neutralization ability of monoclonal antibodies [20,21]. The spike receptor-binding domain (RBD) is the main component for neutralizing antibodies. Multiple antigenic sites are located in the RBD, such as receptor binding site-A (RBS-A), receptor binding site-B (RBS-B), receptor binding site-C (RBS-C), the CR302, and the S309 sites [22]. Further mutation in the omicron spike RBD can locate one or more antigenic sites, which causes resistance of omicron against monoclonal antibodies targeting the sites [23]. A new sublineage of Omicron, BA.2, does not show the S-gene on testing, and additional testing shows that load growth is too large and transmission is widespread. Simply PCR test is used to detect alpha, and beta subvariants but additional changes in S-protein would need to be performed revalidation of mutations to detect the omicron genome [24].

The review work is designed to provide an overview of impact of omicron sub variants in India after second wave of pandemic with Delta variants. Discussion around variant impact on containment and management strategy is also highlighted

## 2. Omicron Subvariants

**Figure 1:**
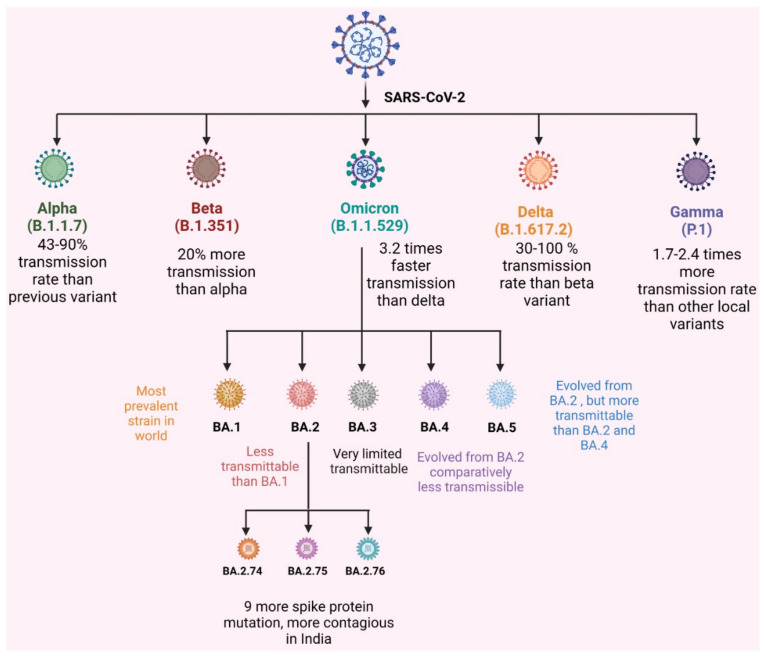
VOCs of SARS-CoV-2 and omicron subvariant transmission rate.

**Figure 2:**
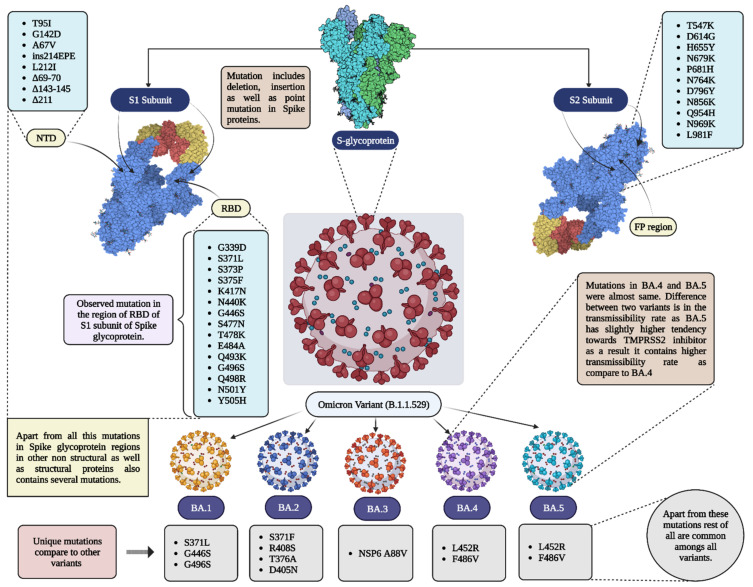
Mutations in different variants of omicron. Pats of spike proteins are shown in 3D models (PDB ID = 7CZQ); RBD = receptor binding domain; NTD = N-terminal domain; FP = fusion protein.

The mutation in the omicron variant (BA.1.1.529) resulted in different sublineages, such as BA.1, BA.2, and BA.3. They were different from one another by differences in S protein mutation, on N- the terminal domain and the receptor binding sites. Later, two other sublineages were identified: BA.4 and BA.5. For the diagrammatic representation of the various variant refer Figure 1. Among them, BA.3 has minor mutations and is not very severe compared to other variants [11]. Detailed information related to mutations in Omicron as well as its subvariants is given in Figure 2 in the form of a schematic illustration.

### 2.1. BA.1

BA.1 was the most productive subvariant exposed worldwide. There were 35 mutations in Omicron resulting in the BA.1 subvariant having 30 amino acid substitutions, three frame deletions and the insertion of amino acids in their spike protein [25]. The mutation in the RBD of the S protein provides a site of the virus to the host cell and targets neutralizing antibodies. Nine of the 15 RBD mutations in the omicron spike region, which is the binding site of the virus to the receptor, human ACE2. The ACE2-RBD binding capacity of BA.1 has the advantage of avoiding detection by neutralizing antibodies (NAbs) [26]. Estimation indicates that omicron BA.1 is 3 to 6 times more contagious and easily spreading than previous variants. Omicron BA.1 has RBD mutations that make it 10 times more infectious than the delta variant. The replication rate of omicron BA.1 is 70% higher than that of the delta aWuhan-Hu-1 variant, but it is less severe in human lung tissues. The secondary attack of the I section was also higher in the BA.1 variant [6]. Tixagevimab/cilgavimab is a monoclonal antibody against the omicron BA.1 variant that neutralizes it.

### 2.2. BA.2

BA.2 has some common mutations of BA.1, but they differ by having specific mutations. BA.2 has additional mutations and lacks the three mutations in BA.1. It has seven exclusive amino acid exchanges. BA.4 and BA.5 have mutations that are closely related to BA.2. An in vitro study on human nasal epithelial cells showed that BA.2 was more contagious than BA.1. cell fusion capacity was also higher in the omicron BA.2 variant [27]. A recent study showed that BA.2 has substitutions such as S371F, T376A, D40, 5 N, and R408S that cause more antibody evasion than BA.1. The primary and booster doses of the CoronaVac inactivated vaccine against BA.2 were 25.1% and 51%, respectively.

### 2.3. BA.4 and BA.5

The availability of BA.4 BA.5 variants were found in people who had been fully vaccinated and showed minor symptoms [28]. The increased rates of BA.4 and BA.5 are associated with their ability to fight against immunity that was developed by past infection, and both variants are capable of breaking out immunological protection that is generated by BA.1. On the other hand, BA.4 and BA.5 cause a new wave of infection due to their capability of neutralizing previous antibodies [29]. BA.5, having greater sensitivity to TMPRSS2 inhibitors, increases the transmission and severity of the disease [30]. In India, the BA.4 and BA.5 variants were less severe, with less than 1% availability. The prevalence of BA.4 and BA.5 lineages is less than 0.5% and 9%, respectively [31].

The study shows that the omicron variant BA.2 BA 2.38, BA 5, BA 2.74, BA 2.75, BA 2.76, and BA. 5 have recently appeared in India. They have acquired additional mutations than BA.2. Among them, BA.2.75 was the predominant omicron sublineage. It has shown a 57-fold higher binding affinity to the ACE2 receptor than BA.5. and causes higher transmission in India [32]. The current study indicates that 92.1% recovery has been observed, and only 4.83% of people required oxygen therapy. The people vaccinated with both doses have higher immune responses, and they are capable of overcoming the BA.2 omicron variant. This shows that BA.2 sublineages cause mild disease in India. However, BA.2.75 has numerous key mutations and has been identified in several states in India [33]. The infection rate is higher than in another subvariant. According to researchers, current vaccines and booster doses do not prevent the infection caused by BA.2.75, but they reduce the risk of severity and morbidity [34] (Figure 1). A study by Wand et al. demonstrated that several mutations in the omicron variant resulted in a reduction in viral S protein neutralization against human convalescent sera [35]. Further details are described in Table 1.

## 3. Impact of Delta and Omicron Variants in India

Five distinct VOCs have been discovered by the WHO [6,44,45]. In particular, a significant rise in COVID-19 occurrences in several Indian states has also been connected to VOCs, including Delta as well as Omicron. Delta variants were discovered in India in October 2020 [9,46,47]. Due to a rare trifecta of alterations (T478K, P681R, and L452R), the delta type, which is particularly aggressive as well as resistant to reducing responses in exposed to infectious or protected individuals, persisted even across the massive group of epidemics in India. Notably, greater incidences of Delta subtype contamination have indeed been associated with S-protein mutations, including D614G, L452R, P681R, and T478K [48,49,50]. With the expansion of its source under subdivisions or sublineages such as AY.1, AY.2, AY.3, and AY.33, Delta has advanced successfully. The delta variation’s AY.1 sublineage carries one more K417 N mutation in its S protein [9]. 

Additionally, the K417 N mutation dramatically improves the antibody avoidance potential. It is already noted that the Beta version contains a K417 N mutation [7,51]. Considering that one of the main hypotheses supporting the reduced vaccination efficiency is associated with conventional S-protein and ACE2, this is crucial for comprehending the function of Delta variant.

The delta species is extremely contagious and 40–60% more contagious than the alpha species. The capacity of the delta strain to trick immunity undoubtedly increases the rate of propagation. Six Indian experts claim that the manifestations of the delta variety, which commonly include heat, sneezing, breathing difficulty, sickness, dysentery, pharyngitis, joint pain, and headaches, are fairly significant [52]. Myalgia, a decrease in the residual taste, tiredness, and rhinorrhoea are additional complaints. Quite readily infecting the recipient’s immunity compared to the original infection, the delta variant has indeed spread very quickly to more than 60 nations. Current estimates indicate that the delta variant contaminated more than 26% of the Indian population, and it also resulted in a decline in vaccine coverage before and after the second pandemic epidemic [53].

The Delta variety very accurately infects the lungs of individuals. Since the variation hits more children and teenagers than earlier versions, incidence rates in these age groups have increased since its publication. The viral load for it is also 1000 times higher than it was after the primary outbreak [54,55]. A Chinese study found that viral loads in illnesses induced through other variants were approximately 1000 times lower than those in outbreaks of the Delta virus [56]. According to the Integrated Disease Surveillance Program (IDSP) of the Indian government, the COVID-19 era’s second wave of health facilities was constructed in tents since more than 32% of people were infected. It has a higher infection rate among anyone under 30 compared to the first wave’s 31%. The disease incidence among those in their 30 s to 40 s was maintained at 21% in both waves. Treatment rates for patients in the 20–39 age range increased from 23.7% to 25.5%, while they increased from 4.2% to 5.8% for patients in the 0–19 age range [57]. The findings also indicated that more patients with identified conditions complained about respiratory problems more frequently throughout this phase. India experienced a rise in COVID-19 cases following the discovery of the delta variant, even though the bulk of cases and fatalities were over 500,000 on 6 May and over 6000 on 9 June, respectively [46]. Before that, the variations that allow Delta to avoid immunization through the previous infection included the decreased neutralizing capability of postoperative serum and vaccine sera against Delta, decreased vaccine efficacy (VE) against transmission, and decreased VE over symptomatic disease following the first-dose vaccine. Furthermore, studies have revealed that Delta has a higher secondary attack rate, growth rate, and reproductive performance. Around India, the Delta wave peaked in the middle of May and lasted approximately three months before quickly decreasing [58].

Low vaccination rates during the Delta pandemic have led to the theory that rising morbidity and mortality rates are to blame for the quick decline in the epidemic. Consequently, 83–86% (1–1/R0) of the community would also need to be immune-based, with a basic reproduction ratio (R0) of 6–7, to stop the Delta pandemic. The aforementioned shows that 53–73% of India’s 1.4 billion people would have contracted Delta within the same three-month period, given a 10–50% sensitivity authorization and a 25–35% high prevalence prior to the Delta wave [59,60]. Ten million COVID-19 cases—or 0.77% of the population—were reported in India. India registered an excess of 19 million instances throughout early March and June 2021, double the proportion anticipated, despite a seven-day state-wide closure that began on 20 April 2021 [61]. Following a four-month reduction, states had an increase in incidence in January 2021. To track the spread of the pandemic virus and find novel variants of concern, the Indian Ministry of Health and Family Welfare announced the establishment of the Indian SARS-CoV-2 Genome Sequencing Consortia Indian SARS-CoV-2 Genomics Consortium (INSACOG) in December 2020 [62]. Considering the negative social, economic, and rehabilitative effects of India’s second citywide lockdown in 2020. PHI conditions include prelockdown intervention strategies used in Maharashtra’s current phase from 28 March to 13 April 2021, and are referred to as exacerbated PHI (nonlockdown) implications [63].

For the duration of the pandemic, several schools in India have been shuttered to stop or restrict virus transmission. Even during the initial pandemic wave, closing schools and attaining better lockdown protocols most likely helped to reduce dispersal. The efficacy of closing schools in India was hotly debated, however, once travel restrictions and physical impediments were lifted. Many individuals think that the significant negative effects on children’s psychological and behavioral growth currently have overtaken the epidemiological advantages of closing schools. Schools reopened in numerous states after the Delta wave in August 2021. Along COVID-19′s worldwide course, several mutations have surfaced [63].

On 26 November 2021, the WHO recognized an altered VOC B.1.1.529, which was discovered in South Africa, as the “Omicron” variant [64]. The first Omicron (BA.1.1) case in Karnataka, India, was confirmed on 3 December 2021. The INSACOG laboratory employed genome sequencing to confirm the findings [65]. During the omicron period, nurses, resident physicians, faculty/scientists, and analytic purposes were the most important staff classifications to demonstrate contamination and eventual illness [66]. The Omicron form spread throughout the planet, not just in Africa. The Omicron variant had a stronger infectious rate than the Delta variant. Because more than 70% of its residents contracted Delta during the second wave, over three-fourths possess hybrid immunity, and India has immunized 95% of its qualified candidates with at least one dose [67]. Finally, Omicron displaced the Delta strain in South Africa, generating a new daily peak of more than 37,000 cases in a record duration of just 20 days. On 7 January 2022, approximately 2.8 million sicknesses occurred globally daily, up from a peak of 0.7 million infections before Omicron [68].

India discovered the BQ.1 subvariant of the omicron after discovering the BF. 7 subvariant in Gujarat. Concurrently, the Maharashtra government issued a COVID-19 increase tricky XBB warning to the public [69]. Aside from XBB, which is a new subvariant of Omicron, the state has reported BQ.1, BA.2.3.20. On 19 January the XE recombinant (BA.1–BA.2) was discovered first in the UK. Mumbai has recorded the first instance of the hybrid mutant strain known as XE, a novel COVID-19 variety. There have been no confirmed occurrences of the XE variant reported in India. The Mumbai local body reported the first instance of the XE variety in March 2022, even though the federal government rejected the findings. Out of the 376 samples examined, 230 Mumbai patients were discovered to have Omicron infection, while one tested positive for the XE variation. The 50-year-old costume designer, whose identity has not been made public, came to Mumbai on 10 February with a filming crew, according to the article. The woman tested positive on 2 March despite having received both doses of the Comirnaty vaccination. Her reports were examined, and it was discovered that she had the COVID XE variant. Her condition was noted in her reports to be asymptomatic without comorbidities [70]. Recombination of the Omicron BA.1 and BA.2 types produced the XE variation. Over 90% of the infections found in 2022 will be caused by the Omicron variation, which has two main subvariants: BA1 and BA.2 [71]. BA.2 was the most prevalent during the third wave in India. Another less popular subvariant is BA.3 [72].

The percentage of COVID-19 infections in Delhi that had the Omicron variant has increased over time (days). A total of 430 (28%) of the 1553 total clinical specimens in December 2021 were infected by Omicron [3]. In the final week of December, 38% of all Delhi samples contained the Omicron variety, which spiked the number of new outbreaks. Thirty-one percent of individuals were still confirmed to be Delta and had additional variations. A total of 433 (50%) of the 863 samples that were gathered between 25 December and 31 December had the Omicron lineage, 293 (34%) had the Delta lineage, and 137 (16%) had other variants. Out of 72 tests conducted in Delhi between 1 and 3 January, 20 (27.7%) specimens were confirmed to be infected with the Delta variant, and 47 (65.3%) samples showed positive results for the Omicron lineage of the new coronavirus. There were just 7% more variations [73,74].

Studies have been performed comparing the growth of the Omicron variation to the rise in new infections in Mumbai. The Brihanmumbai Municipal Corporation (BMC) used the data from the five surveys conducted from the sixth to the tenth. BMC genome sequencing revealed an increase in the percentage distribution of Omicron variations and a decrease in Delta and other variants. At initial periods, the Omicron variety spreads more quickly. The Omicron variation expanded from 2% to 55% in just two weeks and then increased to 89% in just four weeks (January 2020) [75]. After that, the virus’s Omicron variant’s propagation decreased. According to the results of the tenth survey, which was conducted on 3 March. In just 80 days, it went from representing only 2% of all instances to becoming 100% widespread and displacing all other lineages. As per the 3 March 2022 report, Omicron’s frequency increased quickly over time and eventually totally replaced all previous lineages of the virus. After 28 December the increase in instances was even more apparent (1333 cases). Omicron had spread to account for 55% of all outbreaks reported about this time (27 December 2020). The caseload peaked with 20,971 new cases, and the slope then smoothed. Omicron was the dominant variant (89%) according to the results of the eighth wave of genome sequencing performed in Mumbai on 24 January; as a result, the curve flattened. After 8 January, there were 20,318 fewer instances. The parabola flattened on 7 January, although the questionnaire assessment from 24 January validated the Omicron variant’s 89% share. The delay was caused by the time required to analyze the materials and gather the data [76].

India is developing encapsulation techniques for Omicron and delta versions. States should continue to focus on the distribution of new cases and positive diagnostic testing in certain regions to quickly discover COVID-19 hotspots. To aid in rapid diagnosis, states should concentrate on increasing the number of tests and the proportion of RT–PCR testing while striving to attain a positivity rate under 5%. To confirm absorption testing and the timely delivery of samples collected for genome sequencing to designated INSACOG (Indian SARS-CoV-2 Genomics Consortium) labs [77], the national center for disease control, the department of biotechnology (DBT), the government of India, and all “hotspots” must collaborate. The center advised the states to diligently employ the financial help provided by the Indian government under Endoscopic retrograde cholangiopancreatography (ERCP) 1 and 2 or National Center for Diseases (NCDC). There must be enough healthcare facilities dispersed around the state to avoid service disruptions [78].

It is crucial to prepare for and strengthen trained people resources, infrastructure, logistics, and procurement given the pandemic’s global reach. No compromises shall be made in the provision of prompt, high-quality care to patients. Both the total testing rate and the proportion of RT–PCR tests have decreased in some states. Without a sufficient diagnosis, it is exceedingly tedious to determine the exact scope of infection dissemination. States need to upgrade their testing facilities and carefully enforce their testing laws [79]. INSACOG was established to keep track of the country’s circulatory variances. States are required to significantly increase the amount of general population sampling done for genome sequencing by submitting these samples to the INSACOG lab network by the guideline. States and UTs should regularly organize press conferences. During public appearances and public announcements that provide facts, all organizations should proactively and effectively confront systemic problems. The population might also be educated about just the steps taken by the government and prompted to comply with them by adopting COVID-appropriate behaviors and being immunized. States and UTs must uphold the broad “Test-Track-Treat-Vaccinate and COVID-appropriate behavior” philosophy to guarantee the strict application of prevention strategies [80].

## 4. Diagnostic and Treatment Impact

Since the emergence of COVID-19 worldwide, several variants have come across different countries during different periods. Due to high mutation in the spike protein of Omicron variants, enhancement in the spread ability among the population as well as decreased efficacy of various treatment methods against the injection was observed during the pandemic time. In India, during the time of omicron normal as well as specific methods were used for the detection of an infection. The CT scan (X-ray) method is a common method for the diagnosis of COVID infection, including omicron variants, through which we can identify damage in the lungs. Meanwhile, other specific methods were also utilized to identify the proper variant type among all variants of Omicron [81]. As with other variants of COVID-19 for the diagnostic purpose of the Omicron variant rapid test method, as well as polymerase chain reaction (PCR) testing. Specifically, in India during the Omicron wave, the Indian Council of Medical Research (ICMR) approved Omicron kits that work on real-time polymerase chain (RT–PCR) assays. ICMR approved two omicron kits manufactured by two different companies. TATA Medical & Diagnostics Ltd., present in Mumbai Maharashtra, developed an omicron kit named TATA MD CHECK RT–PCR OmiSure (Batch number K035-V001), and another kit is KIRIDA Novus SARS-CoV-2 Real-Time PCR kit- Omicron detection kit (Batch number OD060122/01) manufactured by Kriya Medical Technologies Pvt. Ltd [82]. Various methods given are utilized in India as well as globally. To differentiate subvariants of Omicron from each other and other variants of COVID-19, special techniques are needed. Considering the high-risk factors associated with Omicron patients compared to other viral-infected patients, specific techniques such as molecular diagnosis (nucleic acid-based testing) followed by immunological diagnosis are utilized for proper diagnosis of Omicron variants. Gold standard testing and RT–PCR are other methods that can be utilized for the detection of Omicron infection [83]. Normally, spike glycoprotein is encoded through the S gene, which is most affected due to mutation in the virus [84]. PCR testing and next-generation sequencing (NGS) are molecular diagnosis methods, while antigen and serological testing are immunological diagnosis methods. Generally, during the omicron infection time, antigen, antibody, and nucleic acid-based detection techniques vary from different periods, so for a proper diagnosis of omicron infection, the right method at the right time with proper collection of saliva, nasopharyngeal swabs, throat swabs, and nasal swabs is required [85]. For molecular diagnosis (nucleic acid-based testing), the most preferable sample is a nasopharyngeal swab because of its high sensitivity toward an infection compared to samples that are collected from other sources, such as throat and nasal swabs. As per the research, nasopharyngeal swabs have 97% sensitivity toward detection in molecular diagnostic methods [86]. The PCR method shows results either positive or negative based on the identification of the specific viral RNA of the spike, nucleocapsid, and envelope of the virus structure. Now, in the case of the omicron, which shows high mutations, it creates problems in the COVID test and increases the chances of false results, which ultimately leads to an enhancement in the number of COVID cases. As shown above, the period is also important in the testing. In the first 4 days of post symptom infections, samples contain a high amount of RNA, which makes testing easy and accurate, while after 14 days of post symptom infection, viral RNA decreases in the sample, which makes it difficult to identify the positive patients [87] multitarget assays [88,89] are an excellent option to overcome this issue. In this method to obtain accurate results for distinguishing omicron variants from other variants, specific PCR primers are being developed. Another method named loop-mediated isothermal amplification (LAMP), which is an alternative option to the RT–PCR technique, provides rapid as well as accurate methods because it targets approximately 6 to 8 primer sequences to identify and differentiate variants from other variants [85]. Further clustered regularly interspaced short palindromic repeats (CRISPR) and microarray-based technology are newly developed testing methods utilized for diagnostic purposes [90]. Generation gene sequencing (NGS) is also an accurate and reliable testing method. For the detection of omicron from the whole globe, genome sequences remain the gold standard. This method requires highly trained people as well, and cost wise, it is also very expensive [91].

Enzyme-linked immunosorbent assays (ELISAs), immunochromatographic assays, and immunofluorescence assays are examples of various diagnostic methods based on immunological and antigen-based assays. The focus is on N and S proteins through all these testing methods [13] In contrast with the antigen-based diagnostic methods as well as nucleic acid-based methods, periods for testing are different in the antibody-based diagnostic method. Generally, after 2 weeks of postsymptom infection, the human body starts to produce antibodies that can be detected by ELISA, immunochromatographic assays, and immunofluorescent assays, through which positive (infected person) results can be easily identified [92], which is the main reason for not obtaining proper results of COVID-19 in the early days of infection. Antibodies generated against the viral N and S proteins are the main target of these diagnostic methods. Simple blotting systems and simple testing kits that measure immunoglobulin M (IgM) and IgG ratios in blood samples are some of the techniques that work on the same phenomena [93,94].

After discussing various available diagnostic methods, our next discussion is on the main part, which is as important as testing. During the pandemic as well as currently, various studies and research have been performed on the treatment of COVID-19 along with its different variants. Generally, in India, until the third wave in most of the population development of hybrid immunity was observed [95]. Most of the Indian population was asymptomatic in the Omicron wave, and the majority of the patients with mild symptoms were treated during isolation periods. The Indian government suggests very simple treatment for patients with mild symptoms. In the patients with severe to high risk as well as in serious hospitalized patients, various interleukin-6 receptor blockers and corticosteroids were given owing to their immunomodulatory effects against all variants of omicron. In various patients with omicron, treatment with monoclonal antibodies did not become successful [60,96]. During the pandemic condition in India, patients with omicron were given many antiviral drug therapies. Remdesivir, which inhibits the replication of the viral RNA genome, was approved in 2020 [97]; tocilizumab was approved in 2021 for emergency use [98]; molnupiravir, etesevimab, and bamlanivimab were approved in 2021 [99,100]; and casirivimab and imdevimab were approved in 2021 [101].

On the other hand, many vaccine developers started formulating vaccines against the omicron variants. Nevertheless, no vaccine is available that can give 100% effectivity, but the use of vaccines against omicron patients surely gives preventive action in critical patients. Covishield (P-Spike protein-based vaccine) of the Serum Institute of India (SII) and Oxford-AstraZeneca, which is the first vaccine approved in India, are effective up to 78%–100% in clinical trials. It is given with 2 doses of 0.5 mL at an interval of 3 months for adults [102], ultimately preventing the severity of infection, hospitalization ratio and death number. Another is named Covaxin (inactivated virion-based vaccine) of Bharat Biotech, which is the second vaccine approved in India and is effective up to 93% in severe disease conditions, 78% in symptomatic disease, and 64% in asymptomatic disease conditions during clinical trials. It is given with 2 doses of 0.5 mL at the interval of 28–30 days for adults and adolescents with the same effect as Covishield [103]. Both were initially approved in 2021 for emergency use, but after market authorization, the regulatory authority with certain conditions approves it.

Covovax (Recombinant Protein subunit vaccine) of SII and Novavax Inc. (Gaithersburg, MD, USA) is approved in December 2021 and is 89% effective against Omicron. Corbevax [104] (receptor binding domain-containing protein subunit vaccine) of Biological E Limited, Hyderabad, was the third approved vaccine in India in February 2022. Its overall expective effectiveness is 90% against Omicron. It is given with 2 doses of 0.5 mL at intervals of 28–30 days. ZyCoV-D (plasmid DNA-based vaccine) of Zydus Cadila in partnership with the Department of Biotechnology, Government of India, was approved in August 2021 for emergency use for children and adults above 12 years of age. It is effective up to 66.6% in symptomatic RT–PCR-positive cases. It is given in 3 doses of 2 mg at an interval of 28 days through a needleless jet applicator [105,106].

Sputnik-v (P-Spike protein-based vaccine) of the Russian Direct Investment Fund and Dr. Reddy was approved in April 2021 under emergency use. It is given in 2 doses of 0.5 mL at intervals of 3 weeks to 3 months [107,108]. Another vaccine is Vaxzevria (P-Spike protein-based vaccine) of AstraZeneca, which was approved in 2021 under emergency use. It is given in 2 doses of 0.5 mL at an interval of 3 months [109]. Other approved vaccines include Spikevax (RNA-based vaccine) and Janssen Ad26. COV2. S (Recombinant nonreplicating viral vector-based vaccine present in India. HGCO19 (synthetic type of self-replicating mRNA vaccine) by Genova Biopharmaceuticals, India, is the first omicron-specific Indian vaccine that is currently being developed by the company. It will be given as a booster dose 28 days after the first dose of any COVID vaccine [110]. In addition, BBV154 is the first Indian intranasal vaccine developed by Bharat Biotech, and Hyderabad is currently under development. Currently, BBV154 is under phase 3 clinical trials [111]. These are the various treatment options for omicron variants that were given during the pandemic under the vaccination program.

## 5. Omicron and Vaccination

Omicron is the variant of concern (VOC). It is a highly mutated variant of SARS-CoV-2 that emerged in November 2021 in the southern African continent [112]. It includes BA.1, BA.2, BA.3, BA.4, BA.5 and descendent lineages (Figure 1). These lineages are subvariants of omicron [6]. Global concern has emerged due to the recent emergence of the SARS-CoV-2 variant as omicron. It has spread throughout 77 nations to date [113].

Currently, various vaccines are available for the prevention of SARS-CoV-2 (Table 2). BNT162b2 (Pfizer–BioNTech), ChAdOx1 nCoV-19 (AstraZeneca), and mRNA-1273 (Moderna) vaccines are effective against the omicron variant [114]. BNT162b2 is an mRNA vaccine. It is approved in 149 countries. It is also referred to as Tozinameran. In phase 1, a total of 16 clinical trials, in phase 2, a total of 52 clinical trials, and in phase 3, a total of 29 clinical trials have been performed. ChAdOx1 nCoV-19 nonreplicating viral vector vaccine. It is approved in 149 countries. It is also referred to as AZD1222. A total of 10 phase 1 clinical trials, 39 phase 2 clinical trials, and 22 phase 3 clinical trials have been performed. mRNA-1273 is an mRNA vaccine. It is approved in 88 countries. It is also referred to as elasomeran. A total of 10 phase 1 clinical trials, 36 phase 2 clinical trials, and 24 phase 3 clinical trials have been performed [115]. BNT162b2 is a monovalent vaccination that has been licensed by the US Food and Drug Administration (FDA) for people aged 12 and above, and it is also accessible under emergency use authorization (EUA) for children aged 6 months to 11 years. mRNA-1273 is also monovalent vaccination that has been licensed by the FDA for people over the age of 18 and is accessible under EUA for children aged 6 months to 17 years. In children aged 6 to 17, ChAdOx1 nCoV-19 is immunogenic and well tolerated. According to Bloomberg data, more than 12.7 billion vaccination doses have been administered across 184 countries thus far [116].

**Figure 3:**
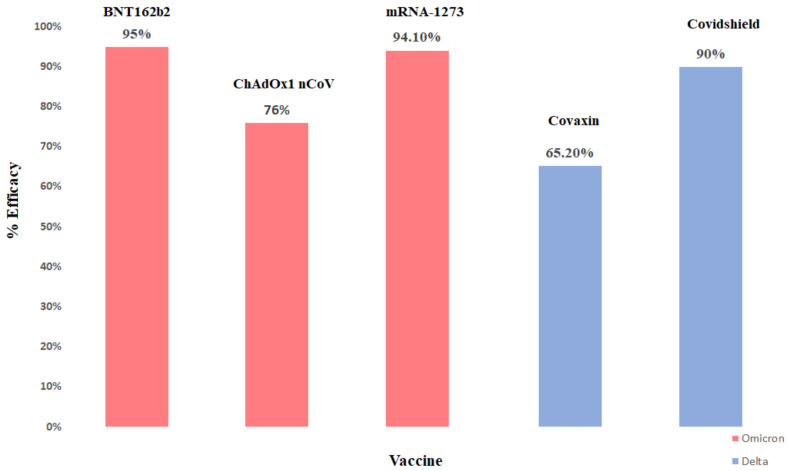
Efficacy rate of different vaccines in India.

There is also evidence that COVID-19 vaccine responses have higher immunization activity at neutralizing BA.1 and BA.2 subvariants of omicron than BA.4/5 subvariants. A total of two doses of BNT162b2 failed to significantly protect against all BA.4/5. It was found that a booster protected BA.4/5, but the shielding may fade after 3 months [123]. Vaccination against symptomatic disease with Omicron 15 weeks after receiving two doses of ChAdOx1 did not protect against symptomatic disease. The effectiveness of the BNT162b2 vaccine was 88.0% 2–9 weeks after receiving the second dose, which was decreased to 48.5% at 10–14 weeks post-dose two and further decreased to 34–37% from 15 weeks post-dose 2. The effectiveness of the ChAdOx1nCoV-19 vaccine as the primary course increased to 71.4% from 2 weeks after a BNT162b2 booster dose. The efficacy of the vaccine increased to 75.5% after the booster dose among those who had received the BNT162b2 primary course [124]. Efficacy of various vaccines towards omicron and delta variant is shown in below Figure 3.

## 6. Vaccine Hesitancy and Current Scenario

A safe and effective vaccination is essential for combating the COVID-19 pandemic [125]. COVID-19 immunization uptake is influenced by vaccine reluctance, socioeconomic variables, and multidimensional deprivation (MPI). A 10% rise in vaccine hesitancy can result in a 30% decrease in vaccination coverage. Similarly, as the fraction of people living in multidimensional poverty rises, so does COVID-19 immunization coverage. The problem of vaccine hesitancy could also be indicated, even among health professionals. One cross-sectional online survey was conducted on nurses between 20 and 28 December 2020 in four different regions of Italy. The online questionnaire was completed by 531 of the 5000 nurses who were invited to participate. Among them, 73.4% were female. A total of 91.5% of nurses intended to accept vaccination, while 2.3% opposed it, and 6.2% were undecided [126]. A unit rise in MPI or the proportion of individuals living in extreme poverty results in a 50% decrease in vaccination coverage. This suggests that increasing socioeconomic hardship has a detrimental impact on health outcomes such as vaccination coverage. The role of gender in vaccine coverage and hesitancy is significant in regard to the use of digital technologies such as the internet. Increased internet access among males leads to an increase in vaccination coverage as well [127]. However, global vaccine distribution remains very uneven at the moment, with the majority of current supplies channeled in the direction of high-income nations [128]. Although the effective and fair distribution of COVID-19 vaccinations is a top governmental objective, guaranteeing acceptance is critical. Trust in vaccinations and the organizations that deliver them is a critical factor in the success of any immunization program. The government of India is pushing all of its citizens to join what is believed to be the world’s largest vaccination campaign, which began on 16 January 2021. Over 219.33 crore Indians have been immunized against COVID-19. In March 2022, COVID-19 immunization was started for children aged 12 to 14 years old. To date, the first dose of COVID-19 vaccination has been given to over 4.11 Cr (4,11,47,585) adolescents. Similarly, COVID-19 booster dosage administration for the age range 18–59 years began on 10 April 2022. Several studies have looked at people’s readiness to take a prospective COVID-19 vaccine in high-income countries, and some have looked into people in middle-income countries as well [129,130,131,132]. However, little is known regarding vaccine acceptability in low-income nations where mass immunization has yet to commence. Compassioning the factors that influence COVID-19 vaccine uptake is of worldwide relevance because a lag in vaccination in any country might result in the establishment and spread of novel variations that can overcome vaccine and previous illness immunity [133,134].

High vaccination reluctance is a serious problem, particularly during the COVID-19 pandemic, and greater efforts should be made to assist individuals and offer correct vaccine information (Table 3).

There is a need to implement educational strategies for health professionals [135].

## 7. Omicron, Monkeypox, and Hand, Foot, and Mouth Disease (HFMD)/Tomato Flu

Omicron has a high infection rate, and the H655Y and N679K mutations near the furin cleavage site can increase spike cleavage, making it more contagious. The 26 amino acid mutations make it distinct from other SARS-CoV-2 variants. It is highly spreading and infectious, and compared to previous alpha, beta, and delta variants, it has 6 or more mutations in its spike protein, the viral ligand that identifies the host cell and binds with the human ACE2 receptor, which targets the body’s immune system. Due to more mutations in the S protein, it blocks the response mediated by T cells and further increases the chances of reinfection [136]. The common symptom of omicron in Indian patients is night sweats, particularly with the BA.5 subvariant [137], loss of smell, and taste are the most commonly reported symptoms, whereas loss of appetite, feeling of fever, cold, cough, myalgia, sore throat, and congestion make it difficult to less damaging variants and other viruses [138].

Monkeypox is a zoonotic virus that causes infection in humans and some animals. Symptoms include fever, swollen lymph nodes, headache, muscle aches, sore throat and cough, and blisters. Monkeypox was first reported in India on 14 July 2022 in Kerala [139]. Monkeypox virus (MPXV) is a double-stranded DNA virus. Zoonotic transmission occurs with direct contact with infected animals and human-to-human transmission through infected respiratory secretions or skin-to-skin contact [140]. Congenital monkeypox is a type in which the virus can transmit to a fetus via the mother’s placenta [141]. As the delta and omicron variant contains an extensively high mutation in the next strain, similarly, the current monkeypox infection was found both geographically and mutationally distant from the probable origin in Nigeria, which is consistent with adaptation by the pathogen [142]. There are two distinct genetic clades of MPXV, the Congo basin clade (clade 1) and the West African clade (clade 2). Clade 2 is further divided into two subclades, 2a and 2b, which are responsible for MPXV infection in 2022 [143]. The major symptom was lymphadenopathy that developed 4 to 10 days after exposure. It is a painful maculopapular rash, lesions are smaller in size and spring water across the face, oral mucosa, palms, and soles. MPXV infection is confirmed by a positive PCR test of the skin. A positive anti-orthodox virus IgM test in serum is suggestive of a recent MPXV infection [144].

A tomato flu reported in India is a variant of hand, foot, and mouth disease (HFMD). Enterovirus 71 is the main causative agent of HFMD. It causes infection mostly in children less than 5 years old [145]. An Indian state of Kerala reported it as ‘tomato flu’. Symptoms of these diseases are tomato-like red spots all over the body, mostly on the hand, foot, and mouth. Other symptoms, such as fever, fatigue, and body aches, are similar to those of COVID-19 [146]. Transmission of virus is facilitated by the environment and by direct contact with an infected person. Diagnosis of HFMD can be achieved from a skin lesion and nasopharyngeal swabs. The rapid diagnostic test for HFMD is RT–PCR [147]. Tomato flu most frequently spreads when people are already affected by COVID-19 and monkeypox. These three infectious viral diseases produced more complications in human health in 2021–2022, especially in India [146].

The third pandemic wave in India was caused by the existence of the Omicron variety, which was present in a negligible number of the country’s total cases as of November 2021. Omicron’s dominance increases quickly over time and eventually supplants all other viral lineages. After contracting omicron, the symptoms often present one to two days later. After December 2021, there was a substantial increase in incidence, with omicron being disseminated in 55% of all infections that were recorded. In the omicron wave, hospital patients were 44 years old on average. Compared to the delta wave, the omicron wave has fewer patients with comorbidities [148]. The time from exposure to the onset of the first symptoms of MPXV was typically 6 to 13 days [149]. A laboratory verified a test for the monkeypox virus using either PCR or sequencing of viral DNA. Communities with high rates of starvation, parasite illness, and other serious health issues are more likely to have MPXV infection [150]. The incubation time of HFMD is 3 to 7 days longer than that of MPXV. While HFMD mostly affected young people, the omicron and MPXV were more severe and were seen in younger and older ages [151].

Omicron viral infection does not have a particular treatment [152]. There was strict self-quarantine as well as medication for minor symptoms. Maintaining hydration and obtaining enough nutrients were important for faster recovery from MPXV infection. antiviral medication Both cidofovir and Brinci-cidofovir are effective in preventing viral DNA polymerase from working. However, vaccinogenic immunoglobulin contributes to postexposure prophylaxis and lessens the disease’s severity [153]. Anti-inflammatory and analgesic drugs used orally or intravenously reduce lymphadenopathy and skin rashes treated with antibiotics. The treatment of human monkeypox in controlled clinical trials, however, has been evaluated [154]. Treatment for HFMD included the topical administration of anesthetics and viscous lidocaine or diphenhydramine for painful mouth ulcers. Fever is treated with antipyretics, and painful ulcers have also been treated with low-level laser treatment [155].

Management of omicron has to be done with the isolation of the infected patients, quarantine, and monitoring of the complication and symptoms. Similar to omicron MPXV management, MPXV management involves patient isolation and protection of compromised skin and mucous membranes. Skin lesions should be covered to the best extent for symptom alleviation, the patient should wear a triple layer mask, and isolation should be continued until all lesions have resolved. For HFMD, prevent direct contact with an infected person. Mild infections do not require hospitalization, and severe symptoms, such as neurological and cardiac complications, require hospitalization. Close observation, careful monitoring, and assessment of fluid balance are important [156].

The death rate of omicron was 81.9% in hospitalized persons among adults and >65 years [157]. In comparison to Omicron, the death rate of MPXV is 0.04% [158]. The study showed that the death rate of HFMD in children 5 years old was 0.78% [159].

## 8. Limitations

There are different diagnoses and treatments available for the management of omicron variants in India. is known to have a severe limitation and risk of transmitting the virus from nasopharyngeal surfaces during molecular testing. Additionally, rapid molecular diagnosis tests increase genetic material in patient samples, making them extremely sensitive, and they may be transmitted if proper sterilization care is not taken during the testing of samples [160]. In India, the most widely used method to determine infection is RT–PCR, which has the limitation that not every RT–PCR can detect the omicron. To screen it, researchers must identify specific sections in virus genetic coding that do not change over time but have features that allow it to be recognized. Further limitation with RT–PCR is that it takes 24 h for detection and results, one has to detect the sequence of virus, which is time consuming as well as expensive, and the biggest issue was having not enough testing kit to meet the broad demands of the pandemic [161]. New mutation in the form of omicron BF.7 is a very quick transfer and has a short incubation period. It is such a horrible scenario that could take place with people traveling across the globe, with this disease spreading worldwide and affecting the direct economy of the nation as well people’s health. This is due to less severeness about vaccination and treatment management, which will lead to increased hospitalization. Genome sequencing should be performed on a minimum of 5% of total positive cases, but especially in India, it has limitations due to the high cost, time consumption and very low number of genome sequencing labs in India [162]. A clinical study was performed to check vaccine effectiveness. A study showed that ChAdOx1 nCoV-19 (AstraZeneca) vaccine effectiveness was lower against the omicron variant. Even after 20 weeks, no effect was noted against the omicron variant at a single dose. Even after two doses, the effectiveness was 65.5%, which is comparatively less than that of other vaccines. As the infectivity of the virus increased, the vaccine rate dropped to 8.8%. Primary immunization with two doses provides limited protection against symptomatic disease caused by the omicron variant [124].

## 9. Future Aspects

It should be important to decide which test should be performed according to the severity of infection and to think about the goal of the test and resources available, as well as test accuracy, accessibility, affordability and speed of detection. It is also suggested that RT–PCR with an SGTF (spike gene target failure) can be used to detect omicrons and will give high outcomes while screening omicrons in India. It is very important to develop primers that are specific for omicrons and will make diagnosis easier, save time and be a more effective technique. It will be beneficial to think about the development of a two-round conformation test to ensure efficient primer binding in current variations. Omicron BF.7 is currently reported in China, so it should be ensured that individuals do not take it lightly, take safety precautions such as wearing masks, quarantine, and follow the required guidelines given by the government. The government should also be making rules to prevent contamination. Researchers should focus on new vaccinations and drug treatments for omicron BFs. to prevent the risk of new variants. Drugs for omicelles can be delivered successfully using nanosystems as a therapeutic alternative.

## 10. Conclusions

Omicron has affected every corner of the world and has a significant impact on India as well. When the world thought of the end of the pandemic, omicron emerged out as a surprise moiety. Because of the high transfer rate, it affected a large population. Currently, no specific treatment against Omicron is available. There has been a significant increase in Omicron cases in India, and several states have already declared additional restrictions to control the new type, despite the fact that it is thought to be milder than the Delta form that wreaked havoc during the second wave. In India, the incidence of infection and transmission is much greater in the third wave than in the second. It does not respond to vaccinated immunity, which demands novel discovery. Vaccination via nasal route and via inhalation may be the better option to get localized IgA based protection and many research around the same are ongoing [21,163,164]. Booster immunizations had no discernible influence on the spread of omicron and deteriorated global vaccine equity. Recently, the Indian government has approved use of Bharat Biotech’s nasal vaccine for people aged 18 and above. The vaccine is available, as a booster dose that may improve the spread via localized immune protection of the upper respiratory track. The diagnosis and treatment of COVID-19 still need to be improved. Although the prevalence of the disease is currently suppressed, it is crucial to study it in detail to build a complete set of management strategies against omicron and other SARS-CoV-2 variants.

## Figures and Tables

**Table 1:** Impact of severity and neutralizing antibody capacity of omicron subvariants.

Sub Variant	Impact on Severity	Neutralizing Antibody	Reference
BA.1	BA. 1 was involved with a lower risk of hospitalization and more severe illness than alpha, beta, and delta variants.	The two doses of BNT162B2 mRNA or coronaVac elicit low neutralizing antibodies in infected persons. However, homologous or heterologous booster doses of these two improve neutralizing antibodies level. 14.2% of the antibody panel, was capable to neutralize BA.1. Antibody germlines IGHV1-58, IGHJ3-1 and IGHV1-69, IGHJ4-1, were used in vaccine protection from BA.1	[36,37,38]
BA.2	BA.2 is less severe than BA.1, and the severity of the variant is decreased in patients with booster dose immunization. Data found that boosters provide 81% of protection against BA.2 Infection.	The neutralizing antibody resistance of BA.2_L452Q was stronger than BA.2_S704 L. 19.9% antibody panel was capable to neutralize BA.2 mAbs EvusheLd(cilagavimab and tixagevimab) can neutralize BA.2 more efficiently than BA.1	[26,39,40]
BA.3	BA.3 was less severe because there was no specific mutations had been observed in its spike protein. It was said changes were due to the combined mutation of BA.1 and BA.2.	Antibody titers against pseudotyped spike protein from ancestral SARS-CoV-2 bearing the D614G mutation.	[41]
BA.4	The BA.4 subvariants increased severity Worldwide because they can spread faster than another variant, but it was less severe than BA.5.	Three doses of BNT162B2 or booster doses of coronaVac/BNT162B2 elicited detectable BA.4 neutralizing antibody responses BA.4 impact against high resistance of antibodies than the previous variant. However, BA.4 is more sensitive to sotrovimab than BA.2	[42]
BA.5	BA.5 variant had to be more transmissible and resistant to adaptive immunity.	BA.5 subvariant would be less susceptible to BNT162b2 or CoronaVac, nevertheless, three doses of BNT162b2 can neutralize antibodies. Same as BA.4, BA.5 4 is more sensitive to sotrovimab than BA.2	[43]

**Table 2:** Various vaccines used for the omicron variants.

Name of Vaccine	Route of Administration	Type of Vaccine	Efficacy	Status	Approval in How Many Countries	Ref.
BNT162b2 (Pfizer–BioNTech)	Intramuscular	mRNA	95.0%	Approved	149	[117,118]
ChAdOx1 nCoV-19 (AstraZeneca)	Intramuscular	Non replicating viral vector	76%	Approved	149	[119,120]
mRNA-1273 (Moderna)	Intramuscular	mRNA	94.1%	Approved	88	[121,122]

**Table 3:** A study of vaccination beliefs and coverage in India.

	Efficacy	Safety Profile	Important for Children	Tuberculosis (BCG)	Diphtheria, Tetanus and Pertussis (DTP1)	Measles (MCV1)	Percent of Parents with a Vaccinated Child	Reference
India	96	97	98	92	94	95	92	[122,123]

## Data Availability

Not applicable.
